# Mitochondrial phylogenies in the light of pseudogenes and *Wolbachia*: re-assessment of a bark beetle dataset

**DOI:** 10.3897/zookeys.56.531

**Published:** 2010-09-17

**Authors:** Wolfgang Arthofer, Dimitrios N. Avtzis, Markus Riegler, Christian Stauffer

**Affiliations:** 1 Molecular Ecology Group, Institute of Ecology, University of Innsbruck, Technikerstrasse 25, 6020 Innsbruck, Austria; 2 NAGREF-Forest Research Institute, 570 06 Vassilika-Thessaloniki, Greece; 3 Centre for Plants and the Environment, School of Natural Sciences, University of Western Sydney, Locked Bag 1797, Penrith South DC NSW 1797, Australia; 4 Institute of Forest Entomology, Forest Pathology and Forest Protection Department of Forest - & Soil Sciences, Boku, University of Natural Resources and Applied Life Sciences, Hasenauerstrasse 38, 1190 Vienna, Austria

**Keywords:** Wolbachia, Scolytinae, pseudogenes, numts, mtDNA, phylogeny, phylogeography

## Abstract

Phylogenetic studies based on mtDNA become increasingly questioned because of potential pitfalls due to mitochondrial pseudogenes and mitochondrial selective sweeps. While the inclusion of nuclear markers should preferentially be considered for future studies, there is no need to abandon mtDNA as long as tests for the known mtDNA artefacts are performed. In this study we presentadditionaldata and test previous phylogeographical studies of Pityogenes chalcographus. We did not detect nuclear copies (numts) of the previously used mitochondrial markers by performing a combined long range/nested PCR of the COI gene and by an in silico analysis of the COI sequence data. This confirms the robustness of our previous phylogenetic study of Pityogenes chalcographus. Results of an in-situ hybridization of Wolbachia in Pityogenes chalcographus confirm the presence of this endosysmbiont in this species. However, we did not detect a correlation between infection status, geographical region and mtDNA haplotypes. The hybridisation data also support a previous hypothesis that infections do not result from parasitoids or parasitic nematodes, insect surface or laboratory contaminations and are hence a true infection of Pityogenes chalcographus. We conclude that the deep structure found in mitochondrial populations of Pityogenes chalcographus indeed represents the evolutionary history of European populations.

## Introduction

In the last two decades several phylogeographic (e.g. Stauffer et al. 1999) and phylogenetic (e.g. Cognato and Sun 2007) studies on scolytines were presented and most of them used mitochondrial DNA (mtDNA) as one of, or the only genetic marker. Analyses of the mitochondrial genome pioneered the era of molecular ecology due to its small size, uniparental mode of inheritance, ease of isolation, and conserved simple structure, allowing the development of universal primers spanning several classes of Metazoa (e.g. Lunt et al. 1999). However, its potential for resolving the evolutionary history of organisms was gradually questioned when factors influencing the reliability of mtDNA derived phylogenies were identified, namely (i) nuclear non-functional copies of mitochondrial genes (e.g. Bensasson et al. 2001), (ii) maternally inherited endosymbionts (Hurst and Jiggins 2005), (iii) positive selection on mitochondrial genomes (Meiklejohn et al. 2007) and (iv) mitochondrial introgression as a consequence of hybridisation (Petit and Excoffier 2009).

Mitochondria originated from the endosymbiosis of α-proteobacteria in ancestral eukaryotic cells. Mitochondrial genomes contain fewer genes than those of free-living α-proteobacteria, due to a loss of genes during their evolutionary history. This gene loss is explained by (1) the functional redundancy of mitochondrial genes with pre-existing nuclear genes and (2) the functional transfer of mitochondrial genes to the nucleus. The transfer of mtDNA derived sequences to the nucleus is an ongoing process in eukaryotes and mitochondrial pseudogenes have been identified in the nuclear genome of many species (Timmis et al. 2004). Such nuclear mitochondrial (numt) pseudogenes can derive from any part of the mtDNA and occur typically as single copies at dispersed genomic locations. Numts are usually less than 1 kb in size (Richy and Leister 2004). Larger fragments as well as tandemly repeated numts have been reported in mammals (e.g. Bensasson et al. 2001). Phylogenies derived solely from mtDNA sequences may hence be erroneous due to numts being co-amplified by universal mitochondrial primers.

A set of strategies is available in order to avoid numt based errors, including in silico analysis of sequences to detect an eventual increased number of non-synonymous base substitutions, frameshifts, additional stop codons and reduced transition/transversion ratios (Bensasson et al. 2001). Positive results should raise doubt on the mitochondrial origin of the retrieved sequences. Furthermore, long PCR techniques can be utilized because most numt sequences are shorter than 1000 base pairs (Richy and Leister 2004).

A specific feature of mtDNA is its strict maternal inheritance in most insects. Due to this asymmetrical inheritance within a species the marker only reflects the female part of the species’ genealogy. Hence, mtDNA transmission will be influenced by any selection for maternally transmitted genes or other maternally selective traits. Several maternally transmitted endosymbionts are well known in invertebrates, with Wolbachia as the most prominent one (Werren et al. 2008). Wolbachia was also detected in Ips typographus (Stauffer et al. 1997), Hypothenemus hamperi (Vega et al. 2002), Xylosandrus germanus (Peer and Taborsky 2005) and Coccotrypes dactyliperda (Zchori-Fein et al. 2006). Recently, Pityogenes chalcographus was found infected with two Wolbachia strains wCha1 and wCha2 (Arthofer et al. 2009a). Both strains occur in low titre not accessible by conventional PCR detection methods.

While some Wolbachia infections do not alter host physiology and reproduction, such effects have been found in others. Reproductive fitness traits range from cytoplasmatic incompatibility (CI) to male-killing, feminisation and the induction of thelytokous parthenogenesis (see Werren et al. 2008 for a review). In a population infected with CI-inducing Wolbachia, the mtDNA associated with the initially infected females will hitchhike through the population and replace the original haplotypes (Hurst and Jiggins 2005). From a phylogenetic point of view this selective sweep may easily be mistaken for a population bottleneck or a founder effect. On the other hand, old and established Wolbachia infections within a population might maintain mitochondrial isolation in spite of nuclear gene flow. In such cases, deep mtDNA structure may contradict homogenous nuclear phylogenies. Thus, the presence of Wolbachia must be checked when mtDNA based phylogenies and phylogeographies are established. This is usally done by conventional PCR using the Wolbachia specific primers for wsp (Zhou et al. 1998) or 16S rDNA (O’Neill et al. 1992). More sophisticated methods include high sensitivity detection (Arthofer et al. 2009a, b) or in situ hybridization which offers a possibility to detect Wolbachia directly in infected tissues (Chen et al. 2005). The latter method reduces the risk of false positive results due to contamination with infected parasitoids, parasitic nematodes or prey in the gut content of predators.

In this study we show that numts do not influence the phylogenetic pattern of Pityogenes chalcographus (Avtzis et al. 2008) by performing a combined long range/nested PCR of the COI gene and by an in silico analysis of the COI sequence data. Furthermore, we present results of an in-situ hybridization of Wolbachia in Pityogenes chalcographus confirming the presence of the endosysmbiont in tissues of this species.

## Material and methods

### Numt search

Mitogenomic sequences of the coleopteran species Pyrocoelia rufa (Lampyridae), Tribolium castanaeum (Tenebrionidae) and Crioceris duodecimpunctata (Chrysomelidae) were obtained from GeneBank (for accession numbers see table 1) and aligned using Clustal X (Thompson et al. 1997). To facilitate identification of conserved regions sequences of Apis mellifera (Apidae), Bombyx mori (Bombycidae) and Drospohila simulans (Drosophilidae) were included in the alignment. Conserved regions were selected for primer design (Table 1). Occasional variable nucleotide positions within the conserved regions required the selection of primer sequences characteristic for coleopterans. Developed primers were Met/F 5’ gctwhtgggttcataccc 3’ located in the methionin tRNA region and CO2/R 5’ caaatttctgaacattg 3’ located in CO2. This primer pair amplifies a stretch of about 3463bp.

**Table 1.** Primer sequences of Met/F and CO2/R for Pityogenes chalcographus amplifying 3463bp: alignments and GenBank accession numbers.

Met/F		5'	g	c	t	w	h	t	g	g	g	t	t	c	a	t	a	c	c	c	3'
Crioceris duodecimpunctata	NC_003372		.	.	.	a	t	.	.	.	.	.	.	.	.	.	.	.	.	.	
Pyrocoelia rufa	NC_003970		.	.	.	t	t	.	.	.	.	.	.	.	.	.	.	.	.	.	
Tribolium castaneum	NC_003081		.	.	.	a	t	.	a	.	.	.	.	.	.	.	.	.	.	.	
Apis mellifera ligustica	NC_001566		.	.	.	a	a	c	a	.	.	.	.	.	.	.	.	.	.	.	
Bombyx mori	NC_002355		.	.	.	a	t	.	.	.	.	c	.	.	.	.	.	.	.	.	
Drosophila simulans	NC_005781		.	.	.	a	c	.	.	.	.	.	.	.	.	.	.	.	.	.	
CO2/R		5'	c	a	a	a	t	t	t	c	t	g	a	a	c	a	t	t	g	3'
Crioceris duodecimpunctata	NC_003372		.	.	.	.	.	.	.	.	.	.	.	.	.	.	.	.	.	
Pyrocoelia rufa	NC_003970		.	g	.	.	.	.	.	.	.	.	.	.	.	.	.	.	.	
Tribolium castaneum	NC_003081		.	.	.	.	.	.	.	.	.	.	.	.	.	.	.	.	.	
Apis mellifera ligustica	NC_001566		.	.	.	.	.	.	.	.	.	.	.	.	.	.	.	.	.	
Bombyx mori	NC_002355		.	.	.	.	.	.	.	.	.	.	.	.	.	.	.	.	.	
Drosophila simulans	NC_005781		.	.	.	.	.	.	.	.	.	.	.	.	.	.	.	.	.	

Fourteen DNA extracts of Pityogenes chalcographus representing all clades were selected for analysis. Thermocycling was performed in a Primus 25 advanced thermocycler (peqlab, Germany). Full length PCR was performed in 10 µl reactions using 0.4 µM of each Met/F and CO2/R primer, 6 mM magnesium sulphate, 200 µM dNTPs, 0.4 U *Taq* DNA polymerase (Sigma, USA), 0.01 U Sawady *Pwo* polymerase (peqlab) and 1 µl DNA template in the buffer provided with the *Pwo* polymerase. Cycling conditions were 3 min initial denaturation at 94° C followed by 32 cycles of 94° C (30 sec), 55° C (1 min) and 68° C (2.5 min) and a final extension step at 68° C (10 min). Products were diluted 1:10,000 with sterile distilled water and 1 µl diluted amplicon was used as template for the nested PCR. Dilution series were carried out to prove that the carry over of genomic DNA from the full length to the nested PCR reaction was small enough to avoid detectable amounts of amplicon. Nested PCR was done in 25 μl reactions containing 3.75 mM magnesium chloride, 125 μM dNTPs (Fermentas, Lithuania), 0.5 μM of each K698 (Caterino and Sperling 1999) and UEA10 (Lunt et al. 1999) primer and 1U Taq polymerase (Sigma, USA). Cycling conditions contained an initial denaturation step of 3 min at 94° C followed by 33 cycles of 94° C (30 sec), 48° C (60 sec) and 68° C (1.5 min) and a final extension step at 68° C (10 min). Amplicon size was checked by gel electrophoresis, products were purified with the QiaQuick PCR purification kit (Qiagen, USA) and Sanger sequencing was performed using nested PCR primers by a commercial provider.

An *in-silco* analysis was performed on 262 sequences of the original study (Avtzis et al. 2008) representing 58 European haplotypes of Pityogenes chalcographus (DQ515997-DQ516054) to identify non-synonymous base substitutions, additional stop codons, insertions and deletions, frameshifts and the transition:transversion ratio. Eleven molecular traits listed in table 2 were selected to discriminate numt and mtDNA which are extensively discussed in the results section.

**Table 2.** *In silico* analysis of CO1 mutations of data presented in Avtzis et al. (2008). Total number and relative amount of mutational patterns observed in a 1557 bp stretch of n=262 individuals is compared with expected values for authentic mtDNA.

	Total	Relative (%)	Expected value for mthNA ^a^
Single base substitutions	125	100.0	-
1^st^ codon position substitutions	15	12.0	14.9 ± 9.4% ^b^
2^nd^ codon position substitutions	2	1.6	4.5 ± 3.5% ^b^
3^rd^ codon position substitutions	108	86.4	80.6 ± 21% ^b^
Nonsynonymous substitutions	13	10.4	7.47 ± 5.4% ^c^
C › T substitutions	25	20.0	-
GC › GT substitutions	3	12.0 ^d^	25 ± 14.0% ^e^
Insertions	0	0	none ^f^
Deletions	0	0	none ^f^
Additional stop-codons	0	0	none ^f^
Transitions (3^rd^ codon position)	95	88.0 ^g^	84.9% ± 18.1% ^h^
Transversions (3^rd^ codon position)	13	12.0 ^g^	15.1 ± 7.6% ^h^
Trasition-transversion ratio	7.31	-	-
GC content	-	34.6	28.66 ± 10.5% ^i^

^a^ expected relative values as given in reference ± χ ^2^ confidence interval at α=0.05 (Sachs 1999), ^b^ Blouin et al. (1998), ^c^ Shoemaker et al. (2004), data of Drosophila subquinaria, ^d^ percentage GC › GT substitutions of total C › T substitutions, ^e^ Bulmer (1986), Bensasson et al. (2001), ^f^ Zhang and Hewitt (1996), ^g^ percentage of total transitions/transversions on 3 ^rd^ codon position, ^h^ Tamura (1992), ^i^ Lin and Danforth (2004), data for CO1 genes

### Identification of Wolbachia infections by *in situ* hybridization

*In situ* hybridization followed a slightly modified protocol of Chen et al. (2005). Insects from locations with elevated Wolbachia prevalence were dissected under a stereo microscope using sterile forceps and scalpel blades. Ovarial tissue was recovered, transferred onto microscope slides, pre-fixed with a drop of methanol and air-dried over night. Final fixation was carried out in a drop of 0.4% formaldehyde at 4° C for 5 min. Slides were washed twice by pipetting 2 ml buffer 1 (100 mM Tris.HCl, 150 mM sodium chloride, pH=7.4) on the tissue. The buffer was kept on the tissue for 30 sec and was then decanted. After 10 min air-drying 10 µl of a hybridization solution containing 1 ng/µl of a DIG-labelled wsp specific probe, 5% (w/v) dextrane sulphate, 2% (v/v) denatured salmon sperm, 1x SSC, 1x Denhart’s reagent and 50% (v/v) formamide were placed on the slide under a cover slip. Tissue was denatured for 5 min at 96° C, cooled on ice and hybridized over night at 42° C in a humid chamber. The cover slip was removed and the slide washed two times 5 min with 2x SSC at room temperature and once 5 min with 0.1x SSC at 42° C. All subsequent steps were carried out at room temperature. The slide was exposed to buffer 2 (100 mM Tris.HCl, 150 mM sodium chloride, 0.5% (w/v) blocking reagent (Roche), pH=7.4) for 15 min, briefly washed with buffer 1 and air-dried for 10 min. 10 µl Anti-DIG antibody conjugated to alkaline phosphatase (Roche, 1:500 in buffer 2) were placed atop each tissue specimen and incubation was performed for one h in a humid chamber. Slides were washed two times 5 min in buffer 1 and equilibrated 5 min in buffer 3 (100 mM Tris.HCl, 150 mM sodium chloride, 1% (w/v) BSA, 0.3% (v/v) Triton X-100, pH=7.4). Staining was performed with 20 µl NBT/BCIP solution (Amresco, USA) in the dark under a cover slide. As soon as a purple colour became visible (30 min up to several h) the cover slip was removed, the sample washed briefly with distilled water, mounted, and microscopy was performed to detect cells infected with Wolbachia. For positive and negative control Drosophila simulans strains were used.

## Results and discussion

Phylogeographic analysis of European Pityogenes chalcographus populations revealed a deep genetic structure between the most diverged haplotypes with three major clades and an estimated divergence time of 100,000 years before present (Avtzis et al. 2008). Recently, low titre infections of two Wolbachia strains were detected in more than 30% of the analysed specimens (Arthofer et al. 2009a). Thus, tests for integrity of the mtDNA based phylogeny in the light of numts and endosymbiont infection were mandatory. Here we present a data set demonstrating that the phylogeny of Avtzis et al. (2008) is not influenced by numt pseudogenes. Arthofer et al. (2009a) have detected Wolbachia in all major Pityogenes chalcographus clades in a pattern that is unlikely to be caused by CI inducing strains. Here we prove the presence of the endosymbiontdirectly in ovarial cells of the beetle, excluding positive Wolbachia detection by PCR due to contamination.

### Long/nested PCR and in silico analysis for presence of numts

Alignment of mitochondrial genomes of three coleopteran and three non-coleopteran insect species resulted in six candidate primers (data not shown), of which one primer pair (Table 1), after extensive optimization of PCR conditions, amplified a clear band from Pityogenes chalcographus DNA extracts. Dilution series of genomic DNA gave no visible bands in dilutions of more than 1:1,000, ensuring that all amplicons produced in the nested PCR originated solely from the full length PCR product and not from genomic carry-over (data not shown). After nested PCR extensive products of the expected size could be obtained from almost all haplotypes of Pityogenes chalcographus examined. Even templates without visible amplification in the full length PCR had formed enough product to be amplified in the subsequent nested reaction. Comparison of the NJ trees derived from direct PCR sequences (Avtzis et al. 2008) and from nested PCR sequences of 14 representative haplotypes of the major clades showed identical topologies (data not shown).

PCR conditions were chosen to remove any numt shorter than 3.4 kb, i.e. three times longer than the largest numts ever observed in insects. Both direct and long/nested PCR sequences were identical, and so were the phylogenetic trees. With our test, co-amplification of numts in the direct PCR approach would have led to discrepancies in tree topology between direct and long PCR sequences.

In order to extend numt screening to 262 individual sequences representing 58 different haplotypes, an in silico analysis was performed targeting characteristic differences between mtDNA and numt sequence composition. Eleven numerical traits were analyzed independently and all of them resulted in values within 5% confidence intervals for authentic mtDNA (Table 2). Thus, presence of numts in the analyzed populations of Pityogenes chalcographus can be excluded.

Several strategies to avoid numt co-amplifications are known. The purification of mtDNA by caesium chloride gradient centrifugation (Nishiguchi et al. 2002) prevents the isolation of numts but is inapplicable when the amounts of source DNA are limited. Beside this, the procedure is slow and laboursome and therefore not suitable for the screening of large populations. Other enrichment techniques provide a DNA that may still be contaminated with some nuclear sequences. In cases where the sequences of authentic mtDNA and the corresponding pseudogenes are known the development of target-specific primers may be recommended (Zhang and Hewitt 1996). The long PCR approach utilized in this study should exclude any amplicons derived from nuclear DNA. Furthermore, mtDNA shows some characteristics in base composition and mutational patterns that are different from the nuclear genome. Most obvious, mtDNA is strongly AT biased (Lewis et al. 1995) and evolves faster than single copy nuclear genes (Galtier et al. 2009). Most probably this fast evolution is explained by inefficient repair mechanisms at the mitochondrial replication complex. More recent studies have shown substantial rate heterogeneity between different species and mitochondrial genes (e.g. Mueller 2006). After transfer into the nucleus, a mitochondrial sequence will evolve with the typical patterns of a pseudogene. Compared to the authentic sequence which is under some selective constraint there will be less codon position bias and a higher proportion of nonsynonymous base replacements (Sunnucks and Hales 1996). Transition-transversion ratio is significantly higher in mtDNA than in corresponding pseudogenes (Arctander 1995). The GC dinucleotide is often methylated in nuclear DNA and 5-methylcytosine mutates abnormally often to T (Bird 1980). Therefore the rate of GC › GT mutations among the four possible nC › nT combinations is highly overrepresented in the nucleus but not in mtDNA where methylation does not occur (Bulmer 1986).

While we consider the long/nested PCR approach as very reliable to exclude any numt from a genetic analysis, it requires additional handling time, costs for PCR consumables and high quality DNA allowing the amplification of >3kb products. Especially the latter condition will not be given when long term stored specimens have to be analyzed that might have degraded DNA. The *in silico* approach presented here can be readily applied to individual haplotypes within any mtDNA alignment and does not require additional manipulations in the laboratory. It is thus suitable for a re-check of existing mtDNA based phylogenies.

### Detection of Wolbachia by *in situ* hybridization

**Figure 1.**
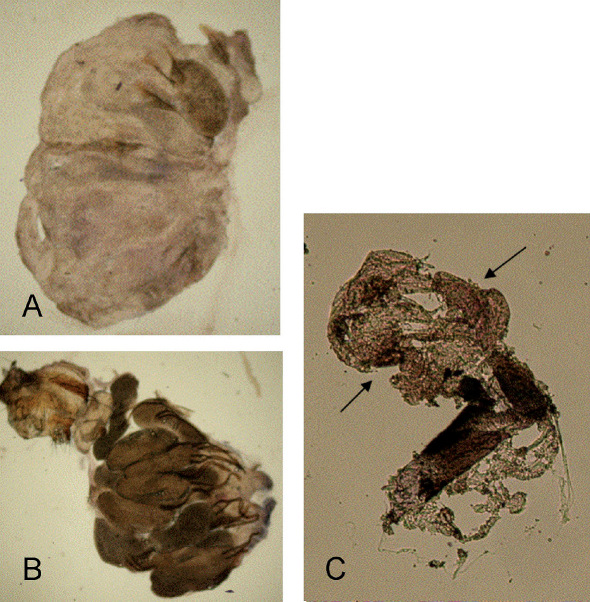
in situ hybridization with wsp specific probe and staining with NBT/BCIP solution on uninfected **A** and Wolbachia infected Drosophila simulans **B**. An accumulation of dark color is observed only in ovarioles of Wolbachia infected Drospohila simulans. **C** Results of in situ hybridization of ovarial tissue excised from one Pityogenes chalcographus individual with accumulation of dark color (arrows). Three specimens were analysed. All ictures taken with 40-fold magnification.

The principial functionality of a modified protocol for Wolbachia detection by in situ hybridization with DIG labelled probes was tested using ovarial tissue of Wolbachia free Drospohila simulans STC and Drospohila simulans flies infected with wRi. Differences in colouration were clearly distiguishable between infected and uninfected Drospohila simulans (Fig. 1 A, B).

Compared to *w*Ri in Drospohila simulans, Wolbachia titre in Pityogenes chalcographus was low, and in average only 35.5% of the individuals were infected (Arthofer et al. 2009a). The ovarial tissue of several individuals analysed showed staining patterns at different intensities, comparable to the Drospohila simulans positive controls (Fig. 1C).

## Conclusion

Evidence of a range of selective forces on mtDNA markers make phylogenetic studies that are purely based on mtDNA less reliable. While the inclusion of nuclear markers like microsatellites or AFLP should preferentially be considered for future studies, there is no need to completely abandon mtDNA as long as tests for the potential manipulation of mtDNA sequences are performed. Such tests should also be included in ongoing efforts to barcode the tree of life based on mtDNA (Song et al. 2008). Here, we confirm that the data of the previous phylogeographic analysis by Avtzis et al. (2008) are not caused by numts. It can be concluded that the deep structure found in mtDNA populations of Pityogenes chalcographus indeed represents the evolutionary history at least of the female branch of European populations

Furthermore, we have detected Wolbachia in Pityogenes chalcographus cells in low titre by in situ hybridisation. Our results confirm earlier work that used a highly sensitive PCR method (Arthofer et al. 2009a). Such an approach can be prone to false positive results due to contamination, as it was found in one extract that carried a uniquely isolated Wolbachia sequence, that most likely derived from co-isolated DNA of a parasitoid (Arthofer et al. 2009a). The previous work showed that two strains are present in this beetle in low titre and low frequency, without any correlation between infection status, geographical region and mtDNA haplotype. Despite the inability to differentiate both strains with the presented hybridisation technique, the new data support that infections do not result from parasitoids, parasitic nematodes or laboratory contaminations and are hence true Wolbachia infections of Pityogenes chalcographus. In general, additional tests for presence of numts and endosymbionts are laborious and time consuming. However they are required for species that exhibit deep mtDNA divergences in order to exclude potential misinterpretation of mtDNA sequence data.
